# Distribution-informed and wavelength-flexible data-driven photoacoustic oximetry

**DOI:** 10.1117/1.JBO.29.S3.S33303

**Published:** 2024-06-05

**Authors:** Janek Gröhl, Kylie Yeung, Kevin Gu, Thomas R. Else, Monika Golinska, Ellie V. Bunce, Lina Hacker, Sarah E. Bohndiek

**Affiliations:** a University of Cambridge, Cancer Research UK Cambridge Institute, Cambridge, United Kingdom; b University of Cambridge, Department of Physics, Cambridge, United Kingdom; c Medical University of Lodz, Department of Biostatistics and Translational Medicine, Poland; d University of Oxford, Department of Oncology, Oxford, United Kingdom

**Keywords:** quantitative imaging, oximetry, deep learning, image processing, simulation

## Abstract

**Significance:**

Photoacoustic imaging (PAI) promises to measure spatially resolved blood oxygen saturation but suffers from a lack of accurate and robust spectral unmixing methods to deliver on this promise. Accurate blood oxygenation estimation could have important clinical applications from cancer detection to quantifying inflammation.

**Aim:**

We address the inflexibility of existing data-driven methods for estimating blood oxygenation in PAI by introducing a recurrent neural network architecture.

**Approach:**

We created 25 simulated training dataset variations to assess neural network performance. We used a long short-term memory network to implement a wavelength-flexible network architecture and proposed the Jensen–Shannon divergence to predict the most suitable training dataset.

**Results:**

The network architecture can flexibly handle the input wavelengths and outperforms linear unmixing and the previously proposed learned spectral decoloring method. Small changes in the training data significantly affect the accuracy of our method, but we find that the Jensen–Shannon divergence correlates with the estimation error and is thus suitable for predicting the most appropriate training datasets for any given application.

**Conclusions:**

A flexible data-driven network architecture combined with the Jensen–Shannon divergence to predict the best training data set provides a promising direction that might enable robust data-driven photoacoustic oximetry for clinical use cases.

## 1 Introduction

Blood oxygen saturation (${\mathrm{sO}}_{2}$) is an important indicator of individual health status used routinely in patient management.^1^ Photoacoustic (PA) imaging (PAI) is a promising medical imaging modality for real-time non-invasive spatially resolved measurement of ${\mathrm{sO}}_{2}$,^2^ with early clinical applications^3^ shown in, for example, inflammatory bowel disease^4^ and cardiovascular diseases.^5^ In cancer, alterations in localized ${\mathrm{sO}}_{2}$ levels have been linked to angiogenesis and hypoxia,^6^ the key hallmarks of cancer that are known to affect treatment outcomes^7^ and are measurable through PAI.

Unfortunately, it remains difficult to apply PAI to derive quantitative values for ${\mathrm{sO}}_{2}$ from multispectral PA measurements.^8^^,^^9^ Linear unmixing (LU) remains the *de facto* standard for ${\mathrm{sO}}_{2}$ estimation from PAI measurements^10^ because of its simplicity and flexibility but has well-understood limitations in its applicability and accuracy.^11^ The limitations of LU are significant in the context of artifacts arising from: the optical processes (e.g., non-linear light fluence distribution^12^ leading to spectral coloring), acoustic processes (e.g., reflection artifacts^13^), or reconstruction algorithms (e.g., model mismatch in sound speed^14^). Using LU is particularly challenging in the presence of highly absorbing tissues, such as the epidermis,^15^ which can introduce reflection artifacts and a spectral bias that leads to an overestimation of ${\mathrm{sO}}_{2}$, which increases with darker skin tone.^16^

Data-driven unmixing schemes have shown promise to alleviate some of the shortcomings of LU^17^^–^^19^ but suffer from three major drawbacks: (1) inflexibility to receiving different input data after training;^20^ (2) performance determined by the composition of the training dataset;^21^ and (3) limited testing on diverse and representative use cases.^22^ In comparison to LU, data-driven methods are often inflexible regarding the input data after training, impacting generalizability, making them difficult to use, and requiring laborious tailoring to a specific application and imaging system. Thus, data-driven ${\mathrm{sO}}_{2}$ estimation methods can struggle to translate promising findings from *in silico* to *in vivo* data.^23^^,^^24^ The lack of high-quality annotated training data and reliable validation data has made it difficult to implement data-driven methods robustly. One way of tackling this challenge lies in bridging the gap between simulated and actual PA data, exploiting realistic phantoms,^25^ an approach that has recently started to be explored.^26^^–^^28^ Furthermore, many data-driven approaches use single-pixel input spectra for their inversion, as with LU, even though 3D inversions would be preferred for realistic use cases.^29^ Solving the full 3D problem is typically computationally intensive, limiting its success *in vivo*.^30^ To make the inverse problem more tractable without full 3D information, some approaches use priors in the inversion scheme,^31^ differential image analysis,^9^ or multiple measurements with differences in illumination.^32^

In this work, we set out to address the three aforementioned limitations. We improve the flexibility of data-driven ${\mathrm{sO}}_{2}$ estimation using a long short-term memory (LSTM) network that enables input wavelength flexibility. We propose a method to inform the choice of training data, which can either be used to choose the best pre-trained model for a target application or to inform the choice of simulation parameters when creating a training data set to underpin a new model. We test these methods on diverse data sources across simulations, phantoms, small animals, and humans.

## 2 Materials and Methods

**Fig. 1:**
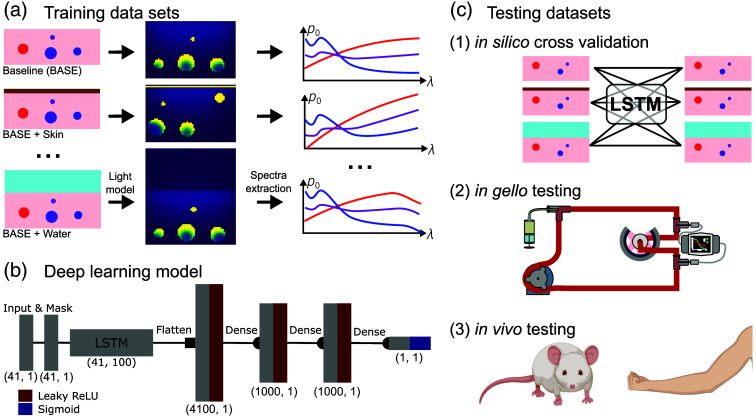
**Overview of the methods used.** (a) Photon transport was simulated with a Monte Carlo light model for each of the 24 distinct datasets adapted from a baseline tissue assumption (BASE). Wavelength-dependent initial pressure spectra (right) were extracted from the vessels simulated in the leftmost panel. (b) A deep learning network based on an LSTM network was introduced to enable greater flexibility regarding input wavelengths for analysis. The hidden state of the LSTM was passed to fully connected layers, which output the estimated blood oxygenation ${\mathrm{sO}}_{2}$. (d) The performance of the LSTM-based method when trained on datasets with different tissue simulation parameters was tested across different datasets, ranging from *in silico* simulations and *in gello* phantom measurements, to *in vivo* measurements. This figure was created with Inkscape using BioRender assets.

We begin by investigating sensitivity to changes in training dataset simulation parameters, by defining a baseline dataset (BASE) with typical assumptions on the tissue geometry and functional parameter ranges, then adapt it into 24 variations [Fig. 1(a)]. We use the Jensen–Shannon divergence to determine the ideal training dataset for a given use case. We propose a deep learning network architecture based on an LSTM network that is flexible regarding the input wavelengths [Fig. 1(b)] and use a testing strategy that comprises computational studies *in silico*, phantoms *in gello*,^33^ and *in vivo* data [Fig. 1(c)].

### 2.1 *In Silico* Datasets

Twenty-five *in silico* datasets were simulated in Python using the SIMPA toolkit^34^ [Fig. 1(a)]. A Monte Carlo model^35^ was used for the optical forward model with a 50 mJ Gaussian beam using $10^{7}$ photons and a 20 mm radius, simulating at 41 wavelengths from 700 to 900 nm in 5 nm steps, and assuming an anisotropy of 0.9. For the datasets that include acoustic modeling, a 2D k-space pseudo-spectral time domain method implemented in the k-Wave toolbox^36^ was used. We assumed a uniform speed of sound of $1500\;{\mathrm{ms}}^{-1}$, a density of $1000\;{g\mathrm{cm}}^{-3}$, and disregarded acoustic absorption. For most datasets, a generic linear ultrasound detector array was placed in the center of the simulated volume. The generic array consists of 100 detection elements (modeled as rectangular elements) with a pitch of 0.18 mm, a length of 0.5 mm, a center frequency of 4 MHz, a bandwidth of 55%, and a sampling rate of 40 MHz. For the datasets mimicking the setup of commercial instruments, we used the built-in device definitions provided in SIMPA.

#### 2.1.1 Baseline dataset simulation parameters

The parameters of the BASE dataset were chosen to reflect the typical parameter choices found in the literature.^20^^,^^29^^,^^37^^–^^40^ A 19.2×19.2×19.2  mm cube was simulated with a 0.3 mm voxel size, resulting in $64^{3}$ voxels per volume. The background started from the second voxel from the top and was modeled as muscle tissue with a blood volume fraction of 1%^20^ with 70% oxygenation.^41^ We added 0 to 9 cylinders with a diameter randomly chosen from U[0.3 mm, 2.0 mm], where U denotes a uniform distribution. The tissue was divided into 3×3 equal-sized compartments in the imaging plane, and a vessel was included with a 50% probability. Each vessel purely contained blood with a randomly drawn ${\mathrm{sO}}_{2}$ value from U[0%, 100%]. To prevent vessel overlap, they were located within their respective compartments’ boundaries.

We defined 24 variations of BASE (Table 1). The variations encompassed a range of tissue backgrounds, vessel sizes, illumination geometries, and resolutions, as well as the addition of a skin layer, performing acoustic simulation, and using digital twins of two commercial instruments (MSOT Acuity Echo and MSOT InVision 256-TF; iThera Medical GmbH, Munich, Germany). The MSOT Acuity Echo is a handheld clinical PAI device with 256 detection elements and an angular coverage of 125 deg, whereas the MSOT InVision 256-TF is a tomographic pre-clinical PAI device with 256 detection elements and a 270 deg view angle. The full details for the digital twin parameters of these devices can be found in prior work^26^^,^^34^ and are available within the SIMPA toolkit. Background heterogeneities were introduced by applying a 3D Gaussian filter of size 1.2 mm to uniform noise.

**Table 1:** Summary of datasets used in the study.

Dataset identifier	Changes from BASE
BG_0-100	Background ${\mathrm{sO}}_{2}$ randomly chosen from U(0%, 100%)
BG_60-80	Background ${\mathrm{sO}}_{2}$ randomly chosen from U(60%, 80%)
BG_H2O	Background modeled as water only
HET_0-100	Background ${\mathrm{sO}}_{2}$ heterogeneously varied between 0% and 100%
HET_60-80	Background ${\mathrm{sO}}_{2}$ heterogeneously varied between 60% and 80%
RES_0.15	Simulation grid spacing: 0.15 mm
RES_0.15_SMALL	Simulation grid spacing: 0.15 mm; radii of vessels halved
RES_0.6	Simulation grid spacing: 0.6 mm
RES_1.2	Simulation grid spacing: 1.2 mm
SKIN	0.3 mm melanin layer; melanosome fraction: U(0.1%, 5%)
ILLUM_5mm	Radius of incident beam: 5 mm
ILLUM_POINT	Radius of incident beam: 0 mm
SMALL	Radii of the vessels halved
ACOUS	Acoustic modeling with linear transducer array
WATER_2cm	2 cm $H_{2}O$ layer added between illumination and tissue
WATER_4cm	4 cm $H_{2}O$ layer added between illumination and tissue
MSOT	Volume extended: 75 × 19.2 × 19.2 mm; MSOT Acuity twin
MSOT_SKIN	MSOT + SKIN
MSOT_ACOUS	MSOT + ACOUS
MSOT_ACOUS_SKIN	MSOT + SKIN + ACOUS
INVIS	Volume extended: 90 × 25 × 90 mm; MSOT InVision 256-tf twin. Single vessel in a 10 mm radius tubular background
INVIS_SKIN	INVIS + SKIN
INVIS_ACOUS	INVIS + ACOUS
INVIS_SKIN_ACOUS	INVIS + SKIN + ACOUS

Each row identifies the changes in dataset creation parameters performed for each of the training datasets relative to the BASE dataset (specified in Sec. 2.1). The left column shows an identifier assigned to each dataset that is used throughout the paper and the right column summarizes the deviation from BASE. U denotes a uniform distribution.

#### 2.1.2 Data preprocessing

We extracted pixel-wise PA spectra from simulations of initial pressure or reconstructed signal amplitude if acoustic simulations were performed. For reconstruction, the backprojection algorithm implemented in the PATATO toolbox was used.^42^ Simulations were processed to extract the spectra in two steps: first, all spectra from blood vessels were selected; second, spectra where the signal intensity at 800 nm was less than 10% of the maximum were discarded. If less than 10% of voxels were chosen this way, we selected the 10% of voxels with the highest signal amplitude from the dataset. We enforced this selection criterion to effectively exclude voxels with low signal-to-noise ratio caused by optical attenuation, as previous works have shown that idealized simulations can contain spectra that display stronger spectral coloring than those actually seen in experimental data from tissues.^20^^,^^32^

The number of extracted spectra from the different dataset variations ranged from 25 thousand to 60 million, compared with BASE with 7 million spectra; the mean over all datasets was just above 6 million. The large difference in extractable spectra is primarily caused by two factors: (1) typical simulations yield 3D $p_{0}$ distributions, but adding acoustic forward modeling leads to a 2D reconstructed image; and (2) some datasets only include a single vessel in the center of the phantom tube, mimicking a blood flow phantom^43^ (described below). To mitigate performance differences caused by this discrepancy, we stratified the dataset sizes by randomly sampling 300,000 spectra with replacement per dataset, which represents a balanced compromise between undersampling larger datasets and oversampling smaller ones.^44^

We performed z-score normalization on each spectrum, setting the mean (μ) to 0 and variance ($\sigma^{2}$) to 1, which discards signal intensity information and eliminates the need for quantitative simulation calibration. Since we performed the same spectra-wise z-score normalization on experimental data, this normalization allows us to apply ${\mathrm{sO}}_{2}$ estimation algorithms trained *in silico* to experimental *in gello* and *in vivo* data.

### 2.2 Deep Learning Algorithm

To address the limited flexibility of data-driven oximetry methods,^20^^,^^40^ a custom unmixing network architecture was composed that contains an LSTM network. Due to the recurrent nature of an LSTM, it can process sparse spectra containing zeros at arbitrary positions [see Fig. 1(b)].

The network input size was fixed at 41, representing the maximum number of wavelengths (between 700 and 900 nm in 5 nm steps) we consider during inference. The number is a trade-off between maximizing the spectral resolution of the input features and simulation time efficiency. The network started with an LSTM layer with a hidden size of 100. A masking layer was used to identify missing values and instruct the LSTM to ignore them. The LSTM output was then flattened into a fixed-length encoding. Following the LSTM, a three-layer fully connected neural network was used with input size 4200, hidden size 1000, and output size 1. A leaky rectified linear unit was used after each layer, and the activation function after the final layer was a sigmoid function to constrain ${\mathrm{sO}}_{2}$ predictions between 0 and 1 [Fig. 1(b)].

The deep learning networks were trained for 100 epochs, where each epoch included the entire training set. The parameters were optimized with the Adam optimizer from the Keras framework^45^ using an initial learning rate of $10^{-3}$ and a mean absolute error loss. The learning rate was halved upon a 5-epoch plateau of the validation loss.

### 2.3 *In Gello* Blood Flow Phantom Imaging

Two variable oxygenation blood flow phantoms were imaged using a previously described protocol.^43^ Briefly, agar-based cylindrical phantoms with a radius of 10 mm were created, and a polyvinyl chloride tube (inner diameter 0.8 mm, outer diameter 0.9 mm) was embedded in the center before the agar was allowed to set at room temperature. The base mixture comprised 1.5% (w/v) agar (Sigma-Aldrich 9012-36-6, St. Louis, Missouri, United States) in Milli-Q water and was heated until dissolved. After cooling to ∼40°C, 2.08% (v/v) of pre-warmed intralipid (20% emulsion, Merck, 68890-65-3) and 0.74% (v/v) Nigrosin solution (0.5  mg/mL in Milli-Q water) were added, mixed, and poured into the mold. Imaging was performed using the MSOT InVision 256TF (iThera Medical GmbH, Munich, Germany) according to a previously established standard operating procedure.^46^ PA images of the phantom were acquired in the range between 700 and 900 nm with 20 nm increments. The first phantom was imaged using deuterium oxide ($D_{2}O$, heavy water) as the coupling medium within the system, while the second was immersed in normal water ($H_{2}O$) during imaging.

### 2.4 *In Vivo* Human Forearm Imaging

Human forearm imaging was performed as part of the PAI Skin Tone study, which started in June 2023 following approval by the East of England—Cambridge South Research Committee (Ref: 23/EE/0019). The study was conducted in accordance with the Declaration of Helsinki and written informed consent was obtained from all study participants. Participants were excluded if they could not give consent, were under the age of 20 or over 80, or had a body mass index outside the range between 18.5 and 30. Imaging was performed using the MSOT Acuity Echo (iThera Medical GmbH, Munich, Germany) using laser light between 660 and 1300 nm, averaging over 10 scans each, and analysis was performed at five wavelengths (700, 730, 760, 800, and 850 nm). One forearm scan from N=7 randomly chosen subjects with Fitzpatrick type 1 or 2 was selected for the purposes of testing the method proposed in this work. The authors manually segmented the radial artery in each scan using the medical imaging interaction toolkit (MITK).^47^

### 2.5 *In Vivo* Mouse Imaging

All animal procedures were conducted under project and personal licenses (PPL no PE12C2B96, PIL no I53057080), issued under the United Kingdom Animals (Scientific Procedures) Act, 1986, and compliance was approved locally by the CRUK Cambridge Institute Biological Resources Unit. Nine healthy 9-week-old female C57BL/6 albino mice were imaged using the MSOT InVision 256TF (iThera Medical GmbH, Munich, Germany) according to a previously established standard operating procedure.^46^ In addition, six 28-week-old healthy female BALB/c nude mice were imaged while inhaling 100% ${\mathrm{CO}}_{2}$ as their terminal procedure. In both cases, imaging was performed at 10 wavelengths equally spaced between 700 and 900 nm averaging over 10 scans each. The mouse body, kidneys, spleen, spine, and aorta were manually segmented by the authors using MITK.

### 2.6 Performance Evaluation

The performance of the LSTM-based method was evaluated using the median absolute error ($\epsilon {\mathrm{sO}}_{2}$) between the estimate (sO^2) and the ground truth/reference ${\mathrm{sO}}_{2}$

ϵsO2=median(|sO2−sO^2|). (1)

Ground truth values are available for the *in silico* and *in gello* datasets. For the *in vivo* measurements, reference values were based on the literature. We assumed ${\mathrm{sO}}_{2}$ of mixed murine blood to be 60% to 70%^41^ and of arterial murine blood ${\mathrm{sO}}_{2}$ under anesthesia to be 94% to 98%.^48^ For the ${\mathrm{CO}}_{2}$ terminal procedure, we assumed that ${\mathrm{CO}}_{2}$ binds to hemoglobin, forming carbaminohemoglobin, which leads to oxygen unloading^49^ and has an absorption spectrum similar to deoxyhemoglobin,^50^^,^^51^ thus continuously decreasing the actual^52^ and measured global blood ${\mathrm{sO}}_{2}$. In humans, arterial blood ${\mathrm{sO}}_{2}$ of 95% to 100% was assumed.^53^

### 2.7 Simulation Gap Measure

To predict the best-fitting training data set for a target application, one could use the ${\mathrm{sO}}_{2}$ estimates of a trained algorithm to calculate error metrics, such as the absolute estimation error, but this is only possible when ground truth or reference ${\mathrm{sO}}_{2}$ values for a representative dataset are available. For *in vivo* applications, this is typically not the case, and unsupervised methods for performance prediction in the context of PA oximetry remain largely unexplored.

Our alternative solution to this problem uses the Jensen–Shannon divergence^54^ ($D_{\mathrm{JS}}$). $D_{\mathrm{JS}}$ measures the distance between distributions and finds application in, e.g., the training of generative adversarial networks.^55^ We compute $D_{\mathrm{JS}}$ between spectra drawn from a reference and a target distribution (i.e., the training and the test data set) and calculate the Pearson correlation coefficient between the resulting $D_{\mathrm{JS}}$ and $\epsilon {\mathrm{sO}}_{2}$ of the LSTM estimates. $D_{\mathrm{JS}}$ measures the distance between unpaired samples drawn from two probability distributions P and Q. $D_{\mathrm{JS}}$ is a symmetric version of the Kullback–Leibler divergence^56^ ($D_{\mathrm{KL}}$) and is defined as

$$D_{\mathrm{JS}}(P||Q)=\frac{1}{2}D_{\mathrm{KL}}(P||M)+\frac{1}{2}D_{\mathrm{KL}}(Q||M),$$ (2)

where $M=\frac{1}{2}(P+Q)$ and the discrete $D_{\mathrm{KL}}$ is defined as the relative entropy between two probability distributions

$$D_{\mathrm{KL}}(P||Q)=\underset{x\in X}{\sum }P(x)\log(\frac{P(x)}{Q(x)}).$$ (3)

To apply these measures, it is important to consider (1) handling the multidimensional probability distributions arising from multi-wavelength measurements and (2) the transformation of two sample distributions into the same sample space. We calculate an aggregate $\bar{D_{\mathrm{JS}}}$ by calculating the mean over the distance for each wavelength in the spectrum

$$\overset{¯}{D_{\mathrm{JS}}}=\frac{1}{N_{\lambda }}\underset{\lambda \in \Lambda }{\sum }D_{\mathrm{JS}}(P_{\lambda }||Q_{\lambda }),$$ (4)

where $N_{\lambda }$ is the number of all available wavelengths Λ. To standardize the sample space, a z-score normalization is performed for each spectrum, and a histogram with 100 bins ranging from −3σ to 3σ is created. A Python implementation of the Jensen–Shannon distance, available in the Scipy (v1.10.1) package,^57^ was used. Using this definition of $\bar{D_{\mathrm{JS}}}$, only the intersection of two different sets of wavelengths can be compared.

## 3 Results

### 3.1 LSTM-Based Method Achieves Accurate Results across a Range of Available Input Wavelengths

**Fig. 2:**
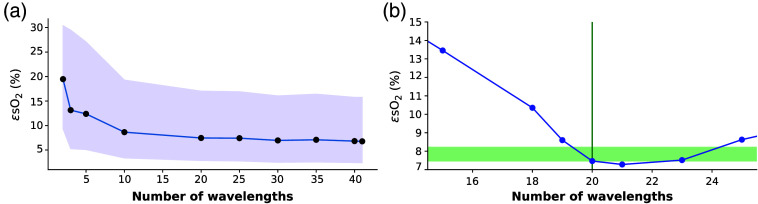
**LSTM-based method shows wavelength flexibility.** (a) LSTMs were trained with varying numbers of wavelengths ($N_{\lambda }$) to show that with an increasing number of wavelengths, the accuracy of the predictions increases. (b) LSTM trained at a given $N_{\lambda }$ can be applied to data with different $N_{\lambda }$ but yields the best results when $N_{\lambda }$ of the test spectra matches that of the training spectra (indicated by the green vertical line).

The accuracy of the LSTM-based network architecture was first tested on the BASE data set, when varying the numbers of available wavelengths ($N_{\lambda }$) for training or inference. $\epsilon {\mathrm{sO}}_{2}$ was extracted when training and testing the network on a certain fixed $N_{\lambda }$, ranging from 3 to 41 wavelengths [Fig. 2(a)]. We found that $\epsilon {\mathrm{sO}}_{2}$ decreased as more wavelengths were used for training. Nevertheless, $\epsilon {\mathrm{sO}}_{2}$ for $N_{\lambda }=10$ was only slightly higher than for $N_{\lambda }=41$ wavelengths. For $N_{\lambda }<10$, $\epsilon {\mathrm{sO}}_{2}$ starts to rapidly increase, which aligns with prior literature^31^ and is potentially exacerbated due to the “vanishing gradient problem”^58^ in LSTMs, which arises when a substantial portion of the input parameter space consists of zeroes.

Next, a network trained at a fixed $N_{\lambda }$ (in this case $N_{\lambda }=20$) was tested on data with a different $N_{\lambda }$ [Fig. 2(b)]. Accuracy was found to decrease rapidly if fewer wavelengths were used for testing, but the error remains low if slightly more wavelengths are used. Nevertheless, the results show that the LSTM-based network performs best if the $N_{\lambda }$ used during inference matches the $N_{\lambda }$ during training.

### 3.2 *In Silico* Cross-Validation Reveals the Effect of Changing Simulation Parameters

**Fig. 3:**
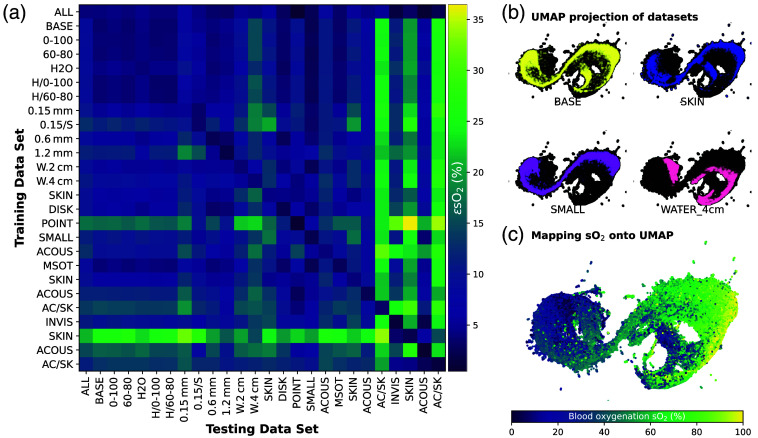
**Dimensionality reduction and cross-validation reveal systematic differences among training datasets.** (a) An LSTM-based network trained on each dataset is then applied to every other dataset, and all $\epsilon {\mathrm{sO}}_{2}$ (median absolute error in percentage points) can be visualized as a performance matrix. Dataset names are shortened for visibility but are detailed in Table 1. (b) UMAP projections of the four representative example datasets onto an embedding of all training data. (c) Mapping the ground truth ${\mathrm{sO}}_{2}$ onto the same projection reveals a correlation along the first UMAP axis.

For each of the 24 simulation parameter variations of BASE, 500 data points with random spatial vessel distributions and a fixed random number generator seed for reproducibility were generated. To investigate the sensitivity of data-driven ${\mathrm{sO}}_{2}$ estimates to changes in training dataset parameters, we first trained LSTM-based networks using all 41 available wavelengths on each simulated training dataset and one on a mixed dataset (ALL). We then performed cross-validation on all datasets by applying every trained network to each of the datasets and calculating the median estimation error $\epsilon {\mathrm{sO}}_{2}$ [Fig. 3(a)]. The $\epsilon {\mathrm{sO}}_{2}$ values range from 0.5% to over 35%. As expected, the best performance occurs when testing on the training set; however, it is important to note that $\epsilon {\mathrm{sO}}_{2}$ is not zero (instead ranging from 0.5% to 5%), suggesting that the network has not overfitted the training data.

We calculated a Uniform Manifold Approximation and Projection (UMAP)^59^ embedding of 200,000 randomly chosen spectra from all datasets. With UMAP, we visualized the location of the spectra of representative datasets on this embedding [Fig. 3(b)]. Examining this visualization indicates that some changes in the dataset parameters result in highly different spectra (e.g., adding a layer of water on top of the tissue), while others lead to only minor variations (e.g., changing the background oxygenation). Labeling the UMAP embedding with the corresponding ground truth ${\mathrm{sO}}_{2}$ values reveals a correlation from low to high oxygenation along the horizontal UMAP axis [Fig. 3(c)].

From the *in silico* cross-validation heatmap [Fig. 3(a)], we can derive several key observations concerning the design of simulated data:

1. Variation in background ${\mathrm{sO}}_{2}$ has a minimal effect with the used 1% blood volume fraction; however, this could become more significant at higher blood volume fractions.
2. Resolution matters: Performance improves with higher spatial resolution simulations in the training data, suggesting that fine details in the spectral data are important for accurate ${\mathrm{sO}}_{2}$ estimation.
3. Illumination matters: When changing from a Gaussian to a point source illumination, the error increased.
4. Chromophore inclusion: When the test dataset contained melanin, but not the training data, the estimation error increased by an average of 5.8 percentage points. When designing a training dataset, all chromophores relevant to the target application should thus be included.
5. Acoustic modeling causes systematic changes: Using acoustic modeling and image reconstruction introduced systematic spectral changes that increase $\varepsilon {\mathrm{sO}}_{2}$ can have detrimental effects on the estimation accuracy and should be considered during training data simulation.
6. Training on a combined dataset is better: Including random samples from all training datasets yielded more accurate estimates for all test datasets *in silico*. It should already be noted that this finding was not reproducible on the experimental datasets, suggesting that the LSTM-based method was not able to generalize better by training on a combined dataset.

### 3.3 Jensen–Shannon Divergence Correlates with the Estimation Error and Can Therefore Be Used to Identify the Best Training Data Set

**Fig. 4:**
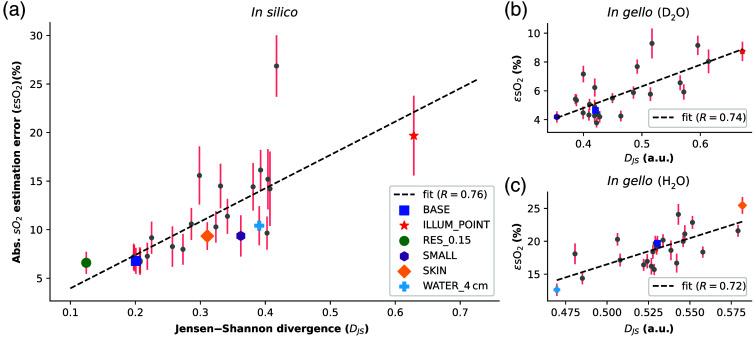
**Jensen–Shannon divergence ($D_{\mathrm{JS}}$) can predict estimation performance.** (a) $D_{\mathrm{JS}}$ correlates with the median absolute ${\mathrm{sO}}_{2}$ estimation error $\epsilon {\mathrm{sO}}_{2}$ when applying all networks, each trained on a distinct training dataset, to the BASE dataset. (b) $D_{\mathrm{JS}}$ for the $D_{2}O$ flow phantom data, which shows a similar correlation with $\epsilon {\mathrm{sO}}_{2}$. (c) After removing two outliers, $D_{\mathrm{JS}}$ shows the same degree of correlation with $\epsilon {\mathrm{sO}}_{2}$ for the $H_{2}O$ flow phantom data.

Given the variance in performance introduced by the choice of training data, it is desirable to automatically determine the best training dataset for a given algorithm and target application. The Jensen–Shannon divergence ($D_{\mathrm{JS}}$) allows one to quantify the distance between the data distribution of each dataset and the target data. We calculated the correlation between $D_{\mathrm{JS}}$ and the median absolute ${\mathrm{sO}}_{2}$ estimation error ($\epsilon {\mathrm{sO}}_{2}$). When applying all networks, each trained on a distinct training dataset, to the BASE dataset, we found that $D_{\mathrm{JS}}$ correlates strongly with $\epsilon {\mathrm{sO}}_{2}$ [Pearson correlation coefficient R=0.76, Fig. 4(a)]. We found the same results when correlating $D_{\mathrm{JS}}$ with the mean squared error (R=0.77). We randomly sampled 100.000 spectra from the entire BASE dataset 10 times and computed the $D_{\mathrm{JS}}$ score for each training dataset. The RES_0.15 dataset, which is the dataset simulated at the highest resolution, and not the BASE dataset, achieved the best $D_{\mathrm{JS}}$ score. A possible reason for this could be that the BASE dataset is a subset of the RES_0.15 dataset and that we drew independent random samples from the entire training distribution.

Extending the analysis to experimentally acquired *in gello* $D_{2}O$ flow phantom data showed a similar correlation (R=0.74). The network trained on SMALL achieved the best score with $D_{\mathrm{JS}}=0.35$ and $\epsilon {\mathrm{sO}}_{2}=4.2\%$ [Fig. 4(b)]. The application of $D_{\mathrm{JS}}$ to the $H_{2}O$ flow phantom experiment initially revealed no correlation (R=−0.1); however, networks trained on ILLUM_POINT and MSOT_ACOUS_SKIN were outliers and after removing these, and the correlation was comparable to other datasets [R=0.72, Fig. 4(c)]. The network trained on WATER_4cm achieved the best score with $D_{\mathrm{JS}}=0.47$ and $\epsilon {\mathrm{sO}}_{2}=12.6\%$. The presence of outliers emphasizes the importance of expert oversight when applying summary metrics such as $D_{\mathrm{JS}}$.

$D_{\mathrm{JS}}$ correlates with $\epsilon {\mathrm{sO}}_{2}$ across multiple simulated and experimental data sets, providing evidence that the Jensen–Shannon divergence can predict algorithm performance. This is particularly relevant for previously unseen datasets where the true ${\mathrm{sO}}_{2}$ is unknown. For each training dataset, a $D_{\mathrm{JS}}$ value can be computed by drawing random samples from both the training and unseen dataset, as outlined in Sec. 2.7. An LSTM-based network pre-trained on the dataset with the lowest corresponding $D_{\mathrm{JS}}$ would then be chosen for data analysis since lower $D_{\mathrm{JS}}$ correlates with a lower $\epsilon {\mathrm{sO}}_{2}$. The same strategy could also be used to guide an optimization process to tailor the simulation parameters to create a new training dataset that matches the target application.

### 3.4 *In Gello* Testing Shows That the LSTM Method Outperforms Learned Spectral Decoloring (LSD)

**Fig. 5:**
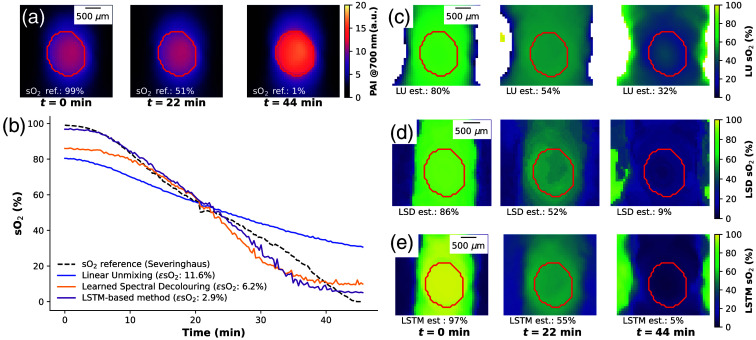
**Estimation of flow phantom data highlights performance dependence on the training dataset.** Three example images of the $D_{2}O$ flow phantom are shown at different time points (0, 22, 44 min) displaying (a) the photoacoustic signal intensity at 700 nm with a red contour marking the blood-carrying tube and (b) the ${\mathrm{sO}}_{2}$ estimations from different methods. We visually compare the performance of LU (c), LSD (d), and the LSTM-based method (e) by plotting the ${\mathrm{sO}}_{2}$ estimations over the same image section and time points shown in panel (a).

Algorithm performance on the oxygenation flow phantom was compared with LU as the *de facto* state of the art and with a previously proposed LSD method.^20^ We show three example PA intensity images at 700 nm of the $D_{2}O$ flow phantom at three time points t = [0 min, 44 min, 70 min] [Fig. 5(a)], annotated with reference oxygenation (${\mathrm{sO}}_{2}^{\mathrm{ref}}$) calculated from ${\mathrm{pO}}_{2}$ reference measurements using the Severinghaus equation.^60^

Comparing the estimates of all methods trained on the SMALL training dataset by plotting the estimated ${\mathrm{sO}}_{2}$ over time [Fig. 5(b)] reveals that the LSTM-based method is, on average, more than twice as accurate as the LSD method and four times as accurate compared with LU. We show the LU estimates for the three example images [Fig. 5(c)], demonstrating the restricted dynamic range of LU estimates from t=0  min to t=44  min, ranging from 80% to 32%, compared with a ground truth of 99% to 1%. Example images for LSD [Fig. 5(d)] and the LSTM-based method [Fig. 5(e)] are shown as well, where the latter can recover the widest dynamic range, extending from 97% to 5%. For both methods, we chose SMALL as the training data set, as it was assigned the lowest $D_{\mathrm{JS}}$ score. We compute the mean over the tube area, outlined in red, to exclude artifacts introduced by the limited transducer bandwidth and the reconstruction algorithm.

### 3.5 Static *In Vivo* Testing Shows the Applicability of the Proposed Method to Different Use Cases

**Fig. 6:**
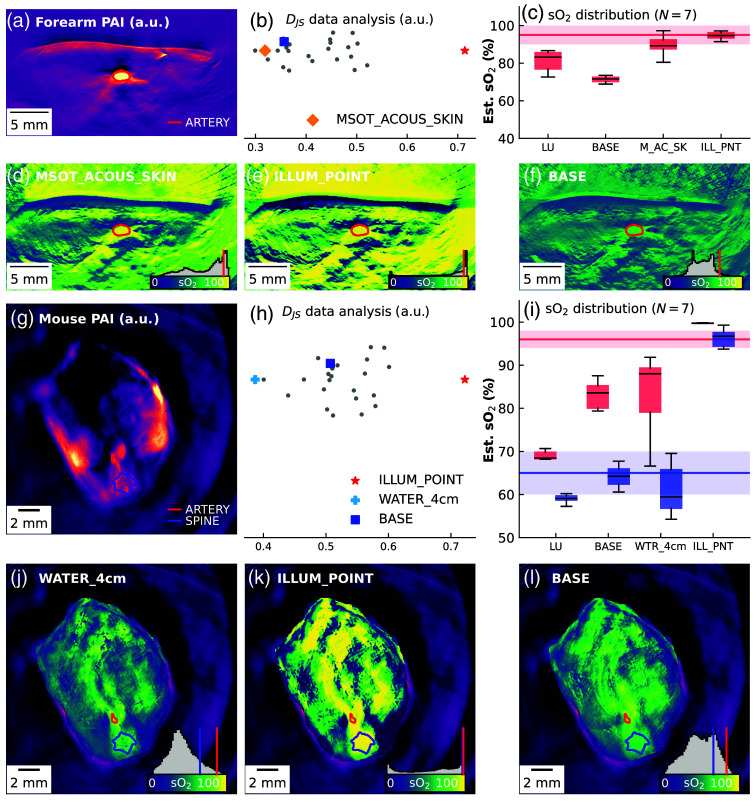
**Jensen–Shannon divergence ($D_{\mathrm{JS}}$) proves valuable for *in vivo* data.** LU and LSTM applied to measurements of the human forearm (a)–(f) and mice abdomens (g)–(l). Panels (a) and (g) show the photoacoustic signal at 800 nm, and panels (b) and (h) show the spread of $D_{\mathrm{JS}}$ estimates for the training datasets. Panels (c) and (i) show boxplots of the highlighted regions of interest over all N=7 subjects. The horizontal lines show expected ${\mathrm{sO}}_{2}$ values for arterial blood (red) and mixed blood (blue). ${\mathrm{sO}}_{2}$ images are shown for models trained on a good fit [(d), (j)], a bad fit [(e), (k)], and the BASE dataset [(f), (l)] as predicted by $D_{\mathrm{JS}}$. On the bottom right of these images, the value distribution is shown as a grey histogram with the mean values of the regions of interest highlighted in their respective color.

Having examined performance in the phantom system with known ground truth, we next apply the data-driven methods to static photoacoustic measurements of seven human forearms [Figs. 6(a)–6(f)] and seven mouse abdomens [Figs. 6(g)–6(l)]. For each, we show an example PA image, calculate $D_{\mathrm{JS}}$ on the data distribution, and compare the estimated blood oxygenation in a region of interest (human forearm: radial artery; mouse: aorta and spine) with literature references.

For the forearm data [Fig. 6(a)], the MSOT_ACOUS_SKIN dataset is objectively the best fit and was assigned the second highest $D_{\mathrm{JS}}$ score, whereas the ILLUM_POINT dataset was the worst fit [Fig. 6(b)]. Some estimated ${\mathrm{sO}}_{2}$ values were close to the expected radial artery ${\mathrm{sO}}_{2}$ value of 95% to 100%. Notably, the network trained on the MSOT_ACOUS_SKIN dataset results in ${\mathrm{sO}}_{2}\approx 90\%$, while the network trained on the ILLUM_POINT dataset produces ${\mathrm{sO}}_{2}\approx 95\%$ [Fig. 6(c)].

The ${\mathrm{sO}}_{2}$ estimates of the network trained on the MSOT_ACOUS_SKIN dataset [Fig. 6(d)] seem to have three primary modes: high values >80% from the vessel structures, values in the 60% to 80% range in the surrounding tissue, and low values from 10% to 50% in the skin and deep tissue. The ${\mathrm{sO}}_{2}$ estimates of the network trained on the ILLUM_POINT dataset [Fig. 6(e)], on the other hand, are concentrated on high ${\mathrm{sO}}_{2}$ values in all superficial tissue and only seem to be below 85% in the skin and in deep tissue. The network trained on the BASE dataset [Fig. 6(f)] estimates low ${\mathrm{sO}}_{2}$ values throughout the entire tissue and does not exceed 80%. The ILLUM_POINT dataset, while seemingly successful if only considering values from the radial artery, was assigned the highest $D_{\mathrm{JS}}$ value. The estimates and marginal histograms show that many estimates are mapped to <20% and >90%, explaining the good score in the radial artery. This finding demonstrates a common limitation of data-driven oximetry methods, where the estimated value distributions do not agree with expectations based on human physiology. The combination of all datasets (ALL) results in an extremely low ${\mathrm{sO}}_{2}$ estimate in the radial artery (median ${\mathrm{sO}}_{2}<50\%$), which contradicts the *in silico* cross-validation results and indicates overfitting of the method to the training datasets.

For mouse images, the aorta and the area around the spinal cord are examined [Fig. 6(g)], assuming from literature a physiological arterial ${\mathrm{sO}}_{2}$ of 92% to 98% and for the spinal cord, a mixed arterial and venous blood with ${\mathrm{sO}}_{2}$ of 60% to 70%. WATER_4cm is objectively the best matching dataset and was assigned the highest score according to $D_{\mathrm{JS}}$ [Fig. 6(h)]. All data-driven methods significantly increase the ${\mathrm{sO}}_{2}$ estimate in the aorta and lie within the desired bounds in the spinal cord [Fig. 6(i)]. The BASE [Fig. 6(l)] and WATER_4cm [Fig. 6(j)] datasets estimate a broad distribution and yield a higher ${\mathrm{sO}}_{2}$ estimate in the aorta and a larger spread between ${\mathrm{sO}}_{2}$ in the aorta and spinal cord compared with LU. The limitations of the ILLUM_POINT [Fig. 6(k)] dataset are even more evident in the mouse data, where even more pixels are either assigned 0% or 100%.

### 3.6 Dynamic *In Vivo* Testing Demonstrates That the LSTM Method Can Reveal Physiological Processes

To provide a quantifiable decrease in the *in vivo* ${\mathrm{sO}}_{2}$ levels in mice that could test the capability of the LSTM-based method, we imaged N=6 mice when experiencing asphyxiation breathing 100% ${\mathrm{CO}}_{2}$.^61^ ${\mathrm{sO}}_{2}$ estimates were extracted from the major visible organs in the scan (spleen, kidneys, spinal cord, and aorta) at 3 min before and 10 min after ${\mathrm{CO}}_{2}$ asphyxiation.

**Fig. 7:**
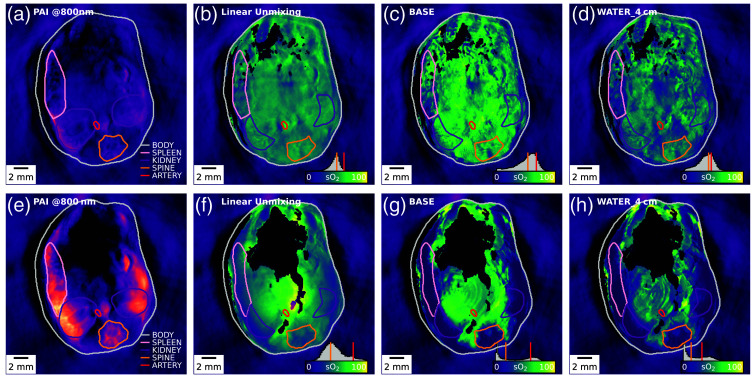
**Data-driven methods estimate an increased ${\mathrm{sO}}_{2}$ dynamic range during ${\mathrm{CO}}_{2}$ delivery compared to LU.** A single representative mouse is shown here. Panels (a)–(d) show the photoacoustic image (a) and ${\mathrm{sO}}_{2}$ estimation results (b)–(d) 3 min *before* asphyxiation, and panels (e)–(h) show the photoacoustic image (e) and ${\mathrm{sO}}_{2}$ estimation results (f)–(h) 10 min *after* asphyxiation. We show ${\mathrm{sO}}_{2}$ estimates for LU [(b), (f)], the BASE [(c), (g)], and the best dataset as predicted by the Jensen–Shannon divergence [WATER_4cm (d), (h)]. Panels (a) and (e) show the outlines of the full-organ segmentations, and all other panels (b)–(d) and (f)–(h) show outlines of the segmented regions used in Table 2.

${\mathrm{CO}}_{2}$ asphyxiation [before, Figs. 7(a)–7(d); after, Figs. 7(e)–7(f)] increases the PA signal amplitude at 800 nm [Figs. 7(a) and 7(e)] in the superficial organs up to a depth of ∼3  mm, while the center of the mouse shows a decrease in signal. Pixels with negative signal intensities at 800 nm were excluded from the analysis (shown in black). ${\mathrm{sO}}_{2}$ values in the examined organs before ${\mathrm{CO}}_{2}$ asphyxiation are generally consistent between LU and data-driven unmixing methods, with the network trained on WATER_4cm estimating slightly lower ${\mathrm{sO}}_{2}$ values (5 to 8 percentage points lower) (Table 2). Notably, the direction of predicted effects on ${\mathrm{sO}}_{2}$ levels aligns well between LU and data-driven unmixing methods. Still, the effect sizes are up to three times greater when utilizing data-driven approaches, demonstrating a wider dynamic range. Intriguingly, in the case of the aorta, LU predicts an increase in ${\mathrm{sO}}_{2}$ levels despite the expected global decrease in ${\mathrm{sO}}_{2}$ levels due to ${\mathrm{CO}}_{2}$ exposure. This may be caused by the aforementioned increase in absorption coefficient in the periphery leading to an increase in spectral coloring in depth.

**Table 2:** ${\mathrm{sO}}_{2}$ decreases during ${\mathrm{CO}}_{2}$ asphyxiation.

Dataset →	LU		BASE		WATER_4cm	
Organ ↓	Before (%)	${\Delta \mathrm{sO}}_{2}$ (%)	Before (%)	${\Delta \mathrm{sO}}_{2}$ (%)	Before (%)	${\Delta \mathrm{sO}}_{2}$ (%)
Body	46±14	−2 (n.s.)	44±20	−7 (**)	37±17	−8 (**)
Spleen	45±10	−10 (**)	40±19	−24 (**)	35±15	−26 (**)
Kidney	52±6	−14 (**)	54±17	−29 (**)	47±11	−32 (**)
Spine	55±6	−14 (**)	60±11	−35 (**)	52±11	−34 (**)
Aorta	67±6	+5 (n.s.)	76±5	−7 (*)	68±17	−18 (*)

Reported are the mean ± standard deviation of ${\mathrm{sO}}_{2}$ measurement before ${\mathrm{CO}}_{2}$ asphyxiation (Before) and the change in the mean (${\Delta \mathrm{sO}}_{2}$) after the procedure. Values are reported at a maximum depth of 3 mm into the mouse body for: LU, an LSTM-based network trained on the baseline training dataset (BASE), and on the best fitting dataset according to the Jensen–Shannon divergence (WATER_4cm). p-values for ${\Delta \mathrm{sO}}_{2}$ are calculated using a non-parametric Mann–Whitney U test and indicated by (n.s. = not significant; *p<0.05; **p<0.01). The segmentation masks are adjusted in Figs. 7(b)–7(d), 7(f)–7(h) to show the region of interest considered when calculating the results.

## 4 Discussion

We present an LSTM-based method for estimating ${\mathrm{sO}}_{2}$ from multispectral PA images. We demonstrate that it can yield superior inference results compared with the previously proposed LSD method while at the same time being usable in a flexible manner, making it a promising candidate to replace LU as the *de facto* state of the art. We also show that the performance of the trained networks is highly dependent on the training data and that changes in simulation parameters can lead to drastically different data distributions. We thus propose to use the Jensen–Shannon divergence ($D_{\mathrm{JS}}$) to complement the LSTM-based method. $D_{\mathrm{JS}}$ correlates with the median absolute ${\mathrm{sO}}_{2}$ estimation error and can thus be used to select the best-fitting training dataset or to optimize the training data distribution to fit the target application.

We highlight how the interplay of the LSTM-based method with $D_{\mathrm{JS}}$ can be used on a diversity of *in vivo* human and mouse data acquired with different scanners and demonstrate that the LSTM-based method can reveal significant dependencies in ${\mathrm{sO}}_{2}$ changes that conventional LU would fail to identify. The LSTM-based method further consistently outperforms LU, with estimated ${\mathrm{sO}}_{2}$ values aligning better with ground truth measurements *in gello* and literature references *in vivo*. LU also shows significant outliers in regions where imaging artifacts are present, which are either not present or less pronounced when using the LSTM-based method.

Our *in silico* cross-validation reveals that acoustic modeling and image reconstruction introduce systematic spectral changes not explained by the initial pressure spectrum alone. Thus, an accurate digital model of the clinically used device is crucial during data simulation to ensure the best algorithm performance. While the combined dataset showed promising results *in silico*, these were not replicated in the experimental datasets, which may suggest that the network is overfitting and able to differentiate between the different simulated datasets.

$D_{\mathrm{JS}}$ appears to be a valuable measure for determining the optimal training dataset for the LSTM-based method, as it correlates with the median absolute ${\mathrm{sO}}_{2}$ estimation error $\epsilon {\mathrm{sO}}_{2}$ *in silico* (R=0.76) and *in gello* (R=0.74/0.72). $D_{\mathrm{JS}}$ predicts a plausible training dataset for all three *in vivo* applications tested in this study, where the predicted value range was 0.4 to 0.7 on the mouse data, 0.5 to 0.8 on the forearm data, and 0.3 to 0.7 on the ${\mathrm{CO}}_{2}$ data. With the development of fast and auto-differentiable simulation pipelines,^62^ it should be possible to optimize the simulation parameters for accurate ${\mathrm{sO}}_{2}$ estimates by iteratively minimizing $D_{\mathrm{JS}}$. When using differentiable implementations of distribution distance measures, it might even be possible to integrate this optimization into an unsupervised training routine.

The *in gello* experiments with $H_{2}O$ as the coupling medium had high $\epsilon {\mathrm{sO}}_{2}$ errors for all ${\mathrm{sO}}_{2}$ estimation methods and $D_{\mathrm{JS}}$ was consistently high. The wavelength-dependent absorption of light by the water couplant likely adds further spectral coloring, which is not present in most of the simulated data sets. The predicted best training dataset was WATER_4cm and the worst ILLUM_POINT, which is consistent with the *in vivo* mouse experiments also having $H_{2}O$ as the coupling medium. Contrary to $D_{\mathrm{JS}}$ prediction, ILLUM_POINT has the lowest $\epsilon {\mathrm{sO}}_{2}$, resulting in no correlation (R=−0.11); after removing outliers, the correlation was on par with the other experiments (R=0.72). In both cases, the estimation error was lower than predicted by $D_{\mathrm{JS}}$; while the distributions were different, the trained networks still managed to estimate accurate ${\mathrm{sO}}_{2}$ values from the training data. Specifically, the ILLUM_POINT dataset, judging from the quasi-bimodal distributions of the network’s estimates on *in vivo* data, appears to have achieved a good agreement with high ${\mathrm{sO}}_{2}$ values purely by chance. More generally for the InVision experiments, the data sets that mimicked the InVision system were not the best-performing according to $D_{\mathrm{JS}}$, indicating greater systematic spectral differences compared with the more generic datasets. Outliers at one extreme, and more subtle impacts of different simulation parameters at another, are obscured by summary measures such as $D_{\mathrm{JS}}$, thus expert oversight for the use and interpretation of summary measures is needed.

The *in vivo* experiments with ${\mathrm{CO}}_{2}$ asphyxiation showed an increase in signal amplitude at 800 nm in the periphery with a decrease in signal in the center of the mouse. These phenomena suggest an overall increase in the absorption coefficient, which could be caused by a range of factors, including blood coagulation,^63^ erythrocyte aggregation,^64^ the presence of bicarbonate ions (${\mathrm{HCO}}_{3}^{-}$) in the blood formed by the dissociation of carbonic acid into bicarbonate and hydrogen ions,^49^ or an increase in blood volume due to blood stasis. Vasoconstriction of the capillaries leading to pallor mortis could also play a role in the better visibility of the superficial organs.^65^

Having applied the LSTM-based method combined with $D_{\mathrm{JS}}$ to a diverse range of data, we believe they provide a promising route to replacing LU for pixel-based PA oximetry. Combining the flexibility in the application of LU with the increase in accuracy of deep learning-based unmixing methods is attractive. The ${\mathrm{CO}}_{2}$ experiment suggests that LU can underestimate the effect size of ${\mathrm{sO}}_{2}$ changes due to its compressed dynamic range and susceptibility to artifacts. We thus recommend that LU should be complemented by a deep learning-based estimation method. The codes and data of this study are available open-source, facilitating its widespread testing and future application.

Nonetheless, there remain limitations to this study that should be the subject of further research. In this work, we investigated $D_{\mathrm{JS}}$ predictions on a predefined set of datasets. Based on the results, we believe that $D_{\mathrm{JS}}$ might be suitable to be used within a minimization scheme to either manually or automatically determine the best choice of simulation parameters for a given data set, but this remains to be investigated.

Low-resolution 2D acoustic modeling was used to limit the computational overhead, yet we found that the acoustic forward model and image reconstruction algorithm introduce systematic changes to the spectra. In the future, high-resolution 3D acoustic simulations that realistically follow the target hardware and model-based image reconstruction algorithms should be conisdered^66^ to limit the influence of artifacts introduced by the simulation or reconstruction algorithms. To account for spectral coloring artifacts more robustly, the full 2D—or better 3D—tissue context should be taken into account within the neural network, as quantitative PAI is only possible with the full 3D context.^12^^,^^21^ In addition, we have shown that good training data are key for deep learning-based methods for PA oximetry, as such, simulating data as realistically as possible is important. One direction toward this is to make use of domain adaptation methods^27^^,^^28^^,^^67^ that adapt simulated training data to appear more realistic.

## 5 Conclusion

The presented LSTM-based approach for ${\mathrm{sO}}_{2}$ estimation from multispectral PA images surpasses the performance of LU and a previously reported data-driven oximetry method, making it a promising candidate to replace LU as the state of the art. We address the impact of training data variations by introducing the Jensen–Shannon divergence ($D_{\mathrm{JS}}$) as a valuable complement, enabling the selection of optimal datasets and fine-tuning for specific applications. Our LSTM-based method consistently outperforms LU, aligning well with ground truth measurements and literature references, while mitigating outliers in regions prone to imaging artifacts. The combination of the flexibility of the novel LSTM-based method with $D_{\mathrm{JS}}$ for training data optimization is a promising direction to make data-driven oximetry methods robustly applicable for clinical use cases.

## Data Availability

The data and code to reproduce the findings of this study are openly available. The data are available under the CC-BY 4.0 license at: https://doi.org/10.17863/CAM.105987. The code is available under the MIT license at: https://github.com/BohndiekLab/LearnedSpectralUnmixing.
